# Supplementary material for: Early Life Adversities and Borderline Intellectual Functioning Negatively Impact Limbic System Connectivity in Childhood: A Connectomics-Based Study
Source: Front Psychiatry. 2020 Sep 11;11:497116. doi: 10.3389/fpsyt.2020.497116 (PMC7518022; doi:10.3389/fpsyt.2020.497116)
Supplement: Supplementary file 1 [file Table_1.docx]

Environmental Stress Check-List (ESCL)

**Name**: ________________________________ **Date of birth**: ____________________________

**Gender**: ________________________________ **Examination date**: ________________________

**Note**: __________________________________________________________________________________

| **DSM V V-code**  **(ICD-10 Z-code)** | **Environmental stressful condition and/or problems** | **Score**  **(1/0)*** |
| --- | --- | --- |
| V61.8 (Z62.891) | Sibling Relational problem |  |
| V61.8 (Z62.29) | Upbringing away from parents |  |
| V61.8 (Z63.8) | High expressed emotion level within family |  |
| V61.20 (Z62.820) | Parent-child relational problem |  |
| V61.29 (Z62.898) | Child affected by parental relationship distress |  |
| V61.03 (Z63.5) | Disruption of family by separation or divorce |  |
| (Z63.2) | Inadequate family support |  |
| V61.21 (Z69.010) | Encounter for mental health services for victim of parental child physical abuse |  |
| V61.21 (Z69.020) | Encounter for mental health services for victim of non-parental child physical abuse |  |
| V61.21 (Z69.010) | Encounter for mental health services for victim of parental child sexual abuse |  |
| V61.21 (Z69.020) | Encounter for mental health services for victim of non-parental child sexual abuse |  |
| V61.21 (Z69.010) | Encounter for mental health services for victim of parental child neglect |  |
| V61.21 (Z69.020) | Encounter for mental health services for victim of non-parental child neglect |  |
| V61.21 (Z69.010) | Encounter for mental health services for victim of parental child psychological abuse |  |
| V61.21 (Z69.020) | Encounter for mental health services for victim of non-parental child psychological abuse |  |
| V62.3 (Z55.9) | Academic or educational problem (Underachievement in school) |  |
| V62.3 (Z55.9) | Academic or educational problem (School-Family conflicts) |  |
| V60.1 (Z59.1) | Inadequate Housing |  |
| V60.2 (Z59.6) | Low income |  |
| V62.4 (Z60.3) | Acculturation difficulty |  |
| V62.4 (Z60.4) | Social exclusion or rejection |  |
|  | Social Services Intervention |  |
|  | Major Psychiatric Diagnosis within the family |  |
|  | Substance abuse within the family |  |

**TOTAL ESCL Score (Sum of single scores)**

* Score 1 if the condition/problem: [1] impacts the diagnosis, course of illness, prognosis, and treatment of a mental disorder AND/OR [2] is part of the focus of treatment AND/OR [3] explains the need for treatment or evaluation AND/OR [4] provides information about circumstances that may impact the care plan.

**Supplementary Table S1. Environmental Stress Check List.**

|  | BIF group | TD group |
| --- | --- | --- |
| V60.1 (Z59.1) - Inadequate Housing | 4 | - |
| V60.2 (Z59.6) - Low income | 6 | - |
| V61.03 (Z63.5) - Disruption of family by separation or divorce | 10 | 1 |
| V61.20 (Z62.820) - Parent-child relational problem | 6 | - |
| V61.21 (Z69.010) Encounter for mental health services for victim of parental child physical abuse | - | - |
| V61.21 (Z69.020) - Encounter for mental health services for victim of non-parental child physical abuse | - | - |
| V61.21 (Z69.010) - Encounter for mental health services for victim of parental child sexual abuse | - | - |
| V61.21 (Z69.020) - Encounter for mental health services for victim of non-parental child sexual abuse | - | - |
| V61.21 (Z69.010) Encounter for mental health services for victim of parental child neglect | 4 | - |
| V61.21 (Z69.020) - Encounter for mental health services for victim of non-parental child neglect | - | - |
| V61.21 (Z69.010) - Encounter for mental health services for victim of parental child psychological abuse | 1 | - |
| V61.21 (Z69.020) - Encounter for mental health services for victim of non-parental child psychological abuse | - | - |
| V61.29 (Z62.898) - Child affected by parental relationship distress | 6 | - |
| 1.V61.8 (*Z*62*.*891) - Sibling Relational problem | 0 | - |
| V61.8 (Z62.29) - Upbringing away from parents | 3 | - |
| V61.8 (Z63.8) - High expressed emotion level within family | 5 | 3 |
| V62.3 (Z55.9) - Academic or educational problem (Underachievement in school) | 29 | - |
| V62.3 (Z55.9) - Academic or educational problem (School-Family conflicts) | 10 | - |
| V62.4 (Z60.3) - Acculturation difficulty | 3 | - |
| V62.4 (Z60.4) - Social exclusion or rejection | 1 | - |
| (Z63.2) - Inadequate family support | 5 | - |
| Social Services Intervention | 5 | - |
| Major Psychiatric Diagnosis within the family | 2 | - |
| Substance abuse within the family | 2 | - |

**Supplementary Table S2 - Environmental Stress Scoring.** Prevalence of Environmental Stress factors in the group of children with Borderline Intellectual Functioning (BIF) and with Typical Development (TD).

| Sub-Network Edges (n=67) and t-values |  |
| --- | --- |
| \| L Anterior Transverse Collateral S \| L Subparietal S \| 3.1 \| \| --- \| --- \| --- \| \| R Subcallosal G \| R Lingual part of the medial occipito-temporal G \| 3.11 \| \| L Cuneus \| R Occipital Pole \| 3.11 \| \| L Subcallosal G \| R Middle Occipital and Lunatus S \| 3.12 \| \| L Superior Temporal S \| R Accumbens \| 3.12 \| \| L Superior Occipital G \| R vPCC G \| 3.13 \| \| L Occipital Pole \| R Temporal Pole \| 3.13 \| \| R vPCC G \| R Calcarine S \| 3.14 \| \| L Accumbens \| R Middle Occipital and Lunatus S \| 3.14 \| \| L Fusiform G \| R Occipital Pole \| 3.14 \| \| L Middle Occipital G \| R Amygdala \| 3.14 \| \| L Superior Temporal S \| R Transverse Frontopolar G and S \| 3.15 \| \| L Collateral and Lingual S \| R Subcallosal G \| 3.15 \| \| L Subcallosal G \| R Collateral and Lingual S \| 3.16 \| \| L ACC G and S \| L Anterior Transverse Collateral S \| 3.17 \| \| L Suborbital S \| L Anterior Transverse Collateral S \| 3.19 \| \| L Putamen \| R Middle Occipital and Lunatus S \| 3.19 \| \| R Middle Occipital G \| R Amygdala \| 3.19 \| \| L Lingual part of the medial occipito-temporal G \| R Occipital Pole \| 3.2 \| \| L pMCC G and S \| L Intraparietal and tansverse parietal S \| 3.23 \| \| R vPCC G \| R Superior occipital and Transverse Occipital S \| 3.23 \| \| L Long Insular G and central S of the Insula \| L pMCC G and S \| 3.24 \| \| L Superior Occipital G \| R Occipital Pole \| 3.24 \| \| R Calcarine S \| R Lingual part of the medial occipito-temporal G \| 3.29 \| \| L Inferior Segment of the Circular S of the Insula \| R Accumbens \| 3.31 \| \| L Collateral and Lingual S \| R Occipital Pole \| 3.32 \| \| L Hippocampus \| R Subcallosal G \| 3.33 \| \| L Anterior Transverse Collateral S \| R Planum polare of the Superior Temporal G \| 3.33 \| \| L Occipital Pole \| R vPCC G \| 3.38 \| \| L Middle Occipital G \| R Pallidum \| 3.38 \| \| R Subcallosal G \| R Middle Occipital G \| 3.4 \| \| L Middle Occipital G \| R Middle Occipital and Lunatus S \| 3.42 \| \| L Anterior Transverse Collateral S \| L Parahippocampal part of the medial occipito-temporal G \| 3.43 \| \| L vPCC G \| L Occipital Pole \| 3.43 \| \| R Subcallosal G \| R Posterior Transverse Collateral S \| 3.48 \| \| L Suborbital S \| R Accumbens \| 3.51 \| \| L ACC G and S \| R Intraparietal and tansverse parietal S \| 3.52 \| \| L Long Insular G and central S of the Insula \| L Anterior Transverse Collateral S \| 3.59 \| \| L Middle Frontal G \| L ACC G and S \| 3.6 \| \| L Middle Occipital G \| R Middle Occipital G \| 3.6 \| \| L Lingual part of the medial occipito-temporal G \| R Pericallosal S \| 3.61 \| \| R Planum polare of the Superior Temporal G \| R Middle Occipital G \| 3.65 \| \| L ACC G and S \| R Collateral and Lingual S \| 3.66 \| \| R vPCC G \| R Superior Occipital G \| 3.68 \| \| L ACC G and S \| R Hippocampus \| 3.68 \| \| R Subcallosal G \| R Middle Occipital and Lunatus S \| 3.75 \| \| L Pericallosal S \| L Calcarine S \| 3.76 \| \| R Subcallosal G \| R Occipital Pole \| 3.76 \| \| L Medial Orbital (Olfactory) S \| L Anterior Transverse Collateral S \| 3.82 \| \| L Medial Orbital (Olfactory) S \| R Middle Occipital and Lunatus S \| 3.85 \| \| L Precuneus \| L Accumbens \| 3.86 \| \| L vPCC G \| R Occipital Pole \| 4.06 \| \| L Middle Occipital and Lunatus S \| R Occipital Pole \| 4.09 \| \| L Anterior Transverse Collateral S \| L Putamen \| 4.12 \| \| L vPCC G \| L Lingual part of the medial occipito-temporal G \| 4.21 \| \| L Orbital (H Shaped) S \| L Anterior Transverse Collateral S \| 4.3 \| \| L Short Insular G \| L Anterior Transverse Collateral S \| 4.31 \| \| L vPCC G \| L Calcarine S \| 4.32 \| \| L Pericallosal S \| R Occipital Pole \| 4.38 \| \| L Middle Occipital G \| R Occipital Pole \| 4.43 \| \| L Pericallosal S \| L Occipital Pole \| 4.49 \| \| L Orbital G \| L Anterior Transverse Collateral S \| 4.7 \| \| L Calcarine S \| R Occipital Pole \| 4.7 \| \| L Calcarine S \| R Pericallosal S \| 4.81 \| \| L Occipital Pole \| R Occipital Pole \| 4.81 \| \| L Occipital Pole \| R Pericallosal S \| 6.07 \| \| R Pericallosal S \| R Occipital Pole \| 6.3 \| | |

**Supplementary Table S3**. **Edges composing the network of statistical differences**. The sub-network presents the following characteristic: number of nodes=51; number of edges=67; p-value=0.045. L=left hemisphere; R=right hemisphere; S=Sulcus/i; G=Gyrus/i; ACC=Anterior Cingulate Cortex; pMCC=Middle-posterior Cingulate Cortex; vPCC=Posterior-ventral part of the Cingulate Cortex. The parcels labeling is the one reported in (1).

**References**

1. Destrieux C, Fischl B, Dale A, Halgren E (2010): Automatic parcellation of human cortical gyri and sulci using standard anatomical nomenclature. *Neuroimage* 53:1-15.
